# Smooth Change, Mechanistic Fluctuation: Thermodynamic System Drift in Protein Evolution

**DOI:** 10.1371/journal.pbio.1001992

**Published:** 2014-11-11

**Authors:** Richard Robinson

**Affiliations:** Freelance Science Writer, Sherborn, Massachusetts, United States of America

If you heat a protein up, the tertiary contacts that secure its three-dimensional shape weaken until eventually the protein unfolds, or “melts.” Not surprisingly, proteins from thermophilic organisms, such as the bacteria that live in hot springs, tend to have higher melting temperatures than their homologs from mesophiles like humans or the bacteria that live within them.

The increased melting temperature of a thermophile protein reflects an increase in the underlying stability of the protein, which itself is the sum of multiple independent properties of both the folded and unfolded states, ultimately encoded in the amino acid sequence of the protein. While differences in melting temperature seem likely to be the product of natural selection, it is less clear that the underlying biophysical differences themselves are the direct result of selection; alternatively, they could arise as a byproduct of other selective events, from neutral sequence drift, or other evolutionary mechanisms. In this issue of *PLOS Biology*, Kathryn Hart, Michael Harms, and colleagues, together with their advisors, Susan Marqusee and Joseph Thornton, show that this alternative explanation is likely the case for at least one protein, a feat they accomplished by measuring the properties of ancient proteins they reconstructed from scratch using statistical evolutionary methods.

The authors began by comparing the optimal growing temperatures of multiple extant bacteria to the melting temperatures of the enzyme RNase H1 (RNH) in each. As expected, an increase in the growing temperature correlated with an increase in the protein melting temperature, strongly suggesting that the melting temperature was selected in response to increasing environmental temperature.

They then traced the evolution of thermostability by statistically reconstructing the protein ancestors of RNH in *Thermus thermophilius* and *Escherichia coli*. The last common ancestor lived 3 billion years ago. Using multiple analytic tools, they reconstructed the sequence of that ancestor's RNH, along with several intermediates on each branch connecting the ancestor to the two modern species. With sequences in hand, they produced each protein, confirming their function by demonstrating that each could degrade RNA.

**Figure 1:**
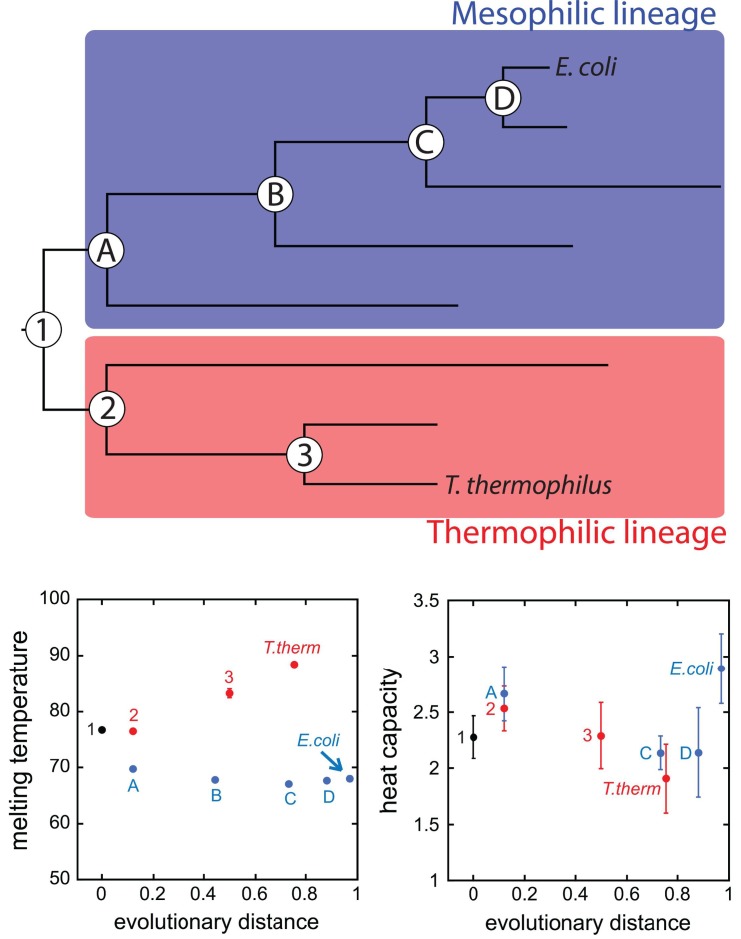
Selection for phenotype does not imply selection for mechanism. Using an evolutionary biophysical approach, Hart et al. revealed that enzyme thermostability evolved smoothly, while the stabilization mechanism fluctuated.

*T. thermophilus*'s RNH melts at 88°C, while *E. coli*'s melts at 68°C. The RNH of the last common ancestor, they found, melted in between, at 77°C. Melting temperatures for the evolutionary intermediates were also intermediate, for the most part, though with a gradual increase along the thermophile branch, but an abrupt decrease early in the mesophile branch (Figure 1).

What accounted for these changes in stability? In previous work, the authors had shown that the difference in the heat capacity of the two modern proteins accounted for more than half the difference in melting temperature between the two. The lower heat capacity of the thermophile's protein is due to its ability to retain residual structure in its unfolded state, they found, an effect they hypothesized might be directly adaptive, since it might minimize irreversible aggregation upon unfolding.

To test whether differences in heat capacity accounted for the differences in melting temperature of the reconstructed ancestral proteins, they determined the free energy of unfolding over a range of temperatures, using a classic thermodynamic equation that relates free energy to three parameters: the heat energy of unfolding, the change in heat capacity upon unfolding as it changes with temperature, and the temperature of maximum stability for the protein. Each of these parameters affects the melting temperature (which appears as an x-intercept in the graph of the equation), and by fitting the parameters to their data, the authors could extract heat capacity, as well as other biophysical information, for each protein.

They found that heat capacity only partly accounted for the increases in melting temperature along the thermophile branch. Contributions were also made by stronger tertiary attractions (reflected in the heat energy of unfolding), and temperature of maximum stability, with the exact mix different for each protein. The same fluctuating pattern of contributions characterized the differences in melting temperatures of proteins along the mesophilic branch as well.

The implication of these results is that while changes in melting temperature itself reflect a response to selective pressure, changes in the underlying mechanisms appear not to be monotonically driven by that same pressure. Within the field of evolutionary development, a fluctuation of underlying mechanism, even while selection directs the outward phenotype, is known as “developmental system drift;” the authors propose the term “thermodynamic system drift” for the analogous process in protein evolution, identified here.

They also point out that the data from their reconstructed proteins complicate the challenge of inferring ancient global temperature patterns based on ancient protein properties. The co-existence in time of a gradual progression in melting temperature of the thermophile branch with the abrupt decline and relative stasis of the mesophile branch suggests that adaptation to local conditions, rather than to any overarching global trend, is a major driver of thermal stability.

**Hart KM, Harms MJ, Schmidt BH, Elya C, Thornton JW, et al. (2014) Thermodynamic System Drift in Protein Evolution.** doi:10.1371/journal.pbio.1001994

